# One-year clinical evaluation of a giomer-based injectable composite compared with a highly viscous glass-ionomer cement in restoring class II cavities of primary molars: a randomized clinical trial

**DOI:** 10.1186/s12903-026-08509-x

**Published:** 2026-05-18

**Authors:** Sarah Emad Ali Eldin, Reham Khaled Elghazawy, Dena Safwat Mustafa, Nagwa Mohamed Ali Khattab

**Affiliations:** 1 Pediatric Dentistry and Dental Public Health Department, Faculty of Dentistry, Ain Shams University, Cairo, Egypt; 2 Operative Dentistry Department, Faculty of Dentistry, Ain Shams University, Cairo, Egypt

**Keywords:** Injectable composite, Glass ionomer cement, Giomer, Class II restorations, Primary molars

## Abstract

**Background:**

Dental caries in the primary dentition remains a major concern, with Class II cavities in molars posing particular restorative challenges. BEAUTIFIL Flow Plus X, a giomer-based injectable composite, offers esthetic and mechanical benefits with fluoride release and easier placement in children, while EQUIA^®^ Forte Fil HT, a glass hybrid restorative, provides enhanced strength for stress-bearing areas. Clinical evidence in primary teeth is still limited, and this study aimed to compare their performance in Class II restorations of primary molars to support material selection in pediatric dentistry.

**Methods:**

A total of 124 primary molars with Class II cavities were randomized with a 1:1 allocation ratio into two groups: Group I received (EQUIA FORTE HT Fil), while Group II received (BEAUTIFIL Flow Plus X F00). Restorations were placed according to manufacturer’s instructions and evaluated for their esthetic, functional, and biological properties using FDI criteria at 3-, 6-, and 12-month intervals. Restorations with total scores of 4 (repair) or 5 (replacement) were recorded as failures. The trial was approved by the Institutional Research Ethical Committee, Faculty of Dentistry, Ain Shams University. Statistical analyses included the Mann-Whitney U test for intergroup and Wilcoxon signed-rank test for intragroup comparisons. The significance level was set at *p* ≤ 0.05.

**Results:**

At 12 months, the clinical success rates were 88.3% for Equia Forte HT Fil and 93.4% for BEAUTIFIL Flow Plus X, with no statistically significant difference (*p* > 0.05). BEAUTIFIL Flow Plus X demonstrated superior esthetic outcomes, with statistically significant advantages in surface luster (*p* = 0.002) and color match (*p* < 0.001). Key functional FDI criteria, including fracture and retention as well as marginal adaptation, were comparable between groups, with no statistically significant differences. Biologically, both materials performed comparably with no significant differences and no observed recurrent caries or postoperative sensitivity in either group.

**Conclusion:**

Both restorative approaches demonstrated acceptable clinical performance in class II cavities of primary molars after 12 months. While functional and biological outcomes were comparable, BEAUTIFIL Flow Plus X provided superior esthetics.

**Trial registration:**

This RCT was registered at Clinical Trial.gov with registration number (NCT06000085) on the date of 11/8/2023.

## Background

Dental caries is one of the most prevalent diseases in the oral cavity, affecting children worldwide, and it remains a significant public health concern [1]. Its high prevalence is linked to inadequate oral hygiene habits, the consumption of carbohydrate-rich food and various socioeconomic and behavioral factors [2–4]. It also significantly affects the oral health-related quality of life of both the child and their family impacting daily function, comfort, and psychosocial wellbeing [5, 6].

Among the different types of caries in primary teeth, proximal caries is prevalent [7, 8]. Its high occurrence can be largely attributed to the morphological characteristics of primary molars, including variations in the contact areas between the first and second primary molars [9, 10]. Other contributing factors include limited salivary access and children’s reduced ability to maintain interproximal oral hygiene, often relying on parental assistance for plaque removal [11, 12]. In addition, difficulty in mechanically controlling biofilm can accelerate the progression of proximal lesions [13] .

Given the widespread occurrence and rapid progression of proximal caries in primary teeth, clinicians are frequently faced with the need for restorative treatment. Restoring Class II lesions represents significant clinical challenges in primary molars [14]. Anatomical characteristics including small crown size, pronounced cervical constriction, thin enamel and dentin make cavity preparation, matrix band placement, and reestablishment of proper proximal contact and contour more difficult [15, 16]. Moreover, the patient’s young age and resistance to dental treatment force the dentist to complete the treatment as quickly and effectively as possible [17]. Therefore, careful material selection and meticulous technique are essential for the long-term success of proximal restorations in primary molars.

Of all materials, Glass-Ionomer Cements (GICs) have gained popularity in pediatric restorative dentistry owing to their unique characteristics. These materials are appreciated for their ease of use owing to their bulk application, which eliminates the high technical sensitivity and time-consuming application compared with conventional composite restorations [18]. However, conventional glass-ionomer cements fell short when used in stress-bearing Class II restorations, primarily due to their inferior mechanical properties [19, 20]. Utilizing glass hybrid technology created a high-strength bulk-fill restorative material, EQUIA^®^ Forte Fil HT, that enhances durability and performance extending the clinical indications to include such applications [21].

Direct resin composites in the posterior region are also increasingly favored as functional and esthetically pleasing restorations. However, similar to all restorative materials, composites have several limitations; the technique sensitivity in the placement procedures, and the occurrence of gap formation that is created by polymerization shrinkage, which can lead to marginal discoloration and secondary caries [22, 23]. To overcome these limitations, flowable composites have been relied on for their simplified placement procedures, and ability to “wet” and conform effectively to the walls and margins of cavities [22, 24]. Advances in filler technology have led to a new generation of flowable composites with higher filler content, commonly referred to as injectable composites. Their flowable consistency allows precise injection and adaptation within the cavity [25].

BEAUTIFIL Flow Plus X is an example of an injectable composite that falls under the category of giomers, which adjoins the beneficial effects of GIC along with the better esthetic and physical characteristics of resin materials. Furthermore, this technology has been reported to prevent demineralization, promote remineralization, inhibit cariogenic bacteria, and exhibit promising clinical behavior and good mechanical properties [26, 27].

Clinicians usually struggle to decide on the restorative material that would provide the best clinical outcomes for their pediatric patients, particularly when managing proximal carious lesions in primary molars. With the continuous emergence of new restorative materials and the scarcity of direct clinical evidence supporting their use in primary teeth, further investigation is warranted. Currently, there is a lack of well-designed randomized clinical trials directly comparing the clinical performance of high-viscosity glass hybrid materials and injectable giomer-based composite restorations in Class II cavities of primary molars. Moreover, limited evidence is available using standardized and internationally accepted evaluation systems, such as the FDI criteria.

Therefore, the present randomized controlled clinical trial was conducted to provide clinical data and to compare the clinical performance of EQUIA^®^ Forte Fil HT and BEAUTIFIL Flow Plus X in restoring Class II cavities in primary molars over a 12-month follow-up period using the FDI evaluation criteria.

The null hypothesis was that there would be no significant difference between EQUIA^®^ Forte Fil HT and BEAUTIFIL Flow Plus X restorations in terms of clinical performance throughout the follow-up period.

## Materials and methods

### Ethical clearance

Ethical approval (FDASU-RecD022210) was obtained from the Institutional Research Ethical Committee, Faculty of Dentistry, Ain Shams University (FDASU-REC) on 16/2/2022. The study was conducted in accordance with the ethical principles outlined in the Declaration of Helsinki. Caregivers of the participating children provided signed informed consent before the commencement of clinical procedures.

### Sample size

Sample size estimation was based on testing the null hypothesis that there is no difference between the two materials in terms of overall clinical performance, expressed as the clinical success rates of the two materials by setting an α error of 5%, a power of 80%, and an allocation ratio of 1:1.The results from previous independent studies suggested that the one-year clinical success rate of the EQUIA Forte in class II restorations in primary molars was 74.4% [28], whereas that for BEAUTIFIL Flow Plus X was estimated to be 94.1% on the basis of the findings of a previous study regarding giomers in class II cavities [29]. A total of 53 primary molars per group were needed. Considering a 15% drop-out rate, the sample size needed was increased to 62 molars per group. Sample size calculation was performed using G*Power software version 3.1.9.4 for MS Windows (Franz Faul, Kiel University, Germany) [30].

### Study design, registration, outcomes

This study was a randomized controlled trial (RCT) with two parallel arms and a 1:1 allocation ratio. The study design adhered to the Consolidated Standards of Reporting Trials (CONSORT 2010) statement. (Additional file 1). It was registered at Clinical Trial.gov with registration number (NCT06000085) in accordance with the International Committee of Medical Journal Editors (ICMGE) consensus statement released in 2004.

The primary outcome of the study was the overall clinical success rate after one year. A restoration was considered clinically successful when all assessed domains received scores ranging from 1 to 3, indicating acceptable clinical performance. This parameter was used for the sample size calculation. The secondary outcomes included the individual FDI criteria within the functional, biological, and esthetic domains which were analyzed to provide a detailed assessment of each material’s performance characteristic. Clinical evaluations were conducted at 3, 6, and 12 months according to the FDI clinical criteria (Additional file 2).

Two hundred fifty children attending the Outpatient Clinic of the Pediatric Dentistry and Dental Public Health Department were screened for eligibility by the principal investigator. This study was performed over a period of 24 months. Screening of patients was carried out during the period from August 2022 till May 2023. The first tooth was enrolled in August 2022 and the last follow up was completed in May 2024.

### Patient eligibility

#### Inclusion criteria

- Four-to eight-year-old children of both sexes.
- Children free from any medical condition that can affect their oral health.
- Cooperative children classified as class 3 or 4 on the basis of Frankl Behavioral Rating Scale, originally developed by Frankl et al. (1962) to classify pediatric dental behavior into four levels ranging from definitely negative to definitely positive [31].

#### Exclusion criteria

- Refusal of the parents to provide informed consent.
- The presence of deep bite or any type of malocclusion or para-functional habits [28].

### Eligibility criteria for teeth

#### Inclusion criteria

- Primary molars with an active proximal caries score of 4 or 5 using ICDAS I [32].
- Primary molars with proximal carious lesions extending in the outer and middle thirds of dentin scored D1 and D2 according to the American Dental Association Caries Classification System (ADA CCS) [33] .
- Simple class II cavities.
- The antagonistic and adjacent tooth should be present.
- The primary molar should have at least 2/3 of the root present.

#### Exclusion criteria

- Signs and symptoms of irreversible pulpitis, such as unprovoked pain, gingival swelling, abscess, or fistula.
- Radiographic signs of any pathological changes.
- Subgingival proximal cavities extend very deep beyond the gingival margin.
- The presence of any physiological or pathological mobility.
- Cavities extend beyond the proximal line angles.

### Randomization and allocation

*Eligible teeth were randomly and equally allocated to the following groups (62 each)*:

Group I, referred to as EQUIA group (EQUIA Forte HT Fil), served as the control group, while Group II, referred to as BEAUTIFIL group (Giomer; BEAUTIFIL Flow Plus X), represented the experimental group.

In participants with multiple eligible carious primary molars, only one tooth was selected to be treated with either the EQUIA group or the BEAUTIFIL group. The patients were allowed to draw from number of cards corresponding to the number of their eligible teeth. This was done to alleviate the clustering effect and minimize the potential influence of multiple confounding factors on the included restorations. This effect arises because teeth within the same individual are more likely to share similar characteristics or outcomes, leading to correlated data [34].

After selecting one tooth per participant, treatment allocation for the selected tooth was determined using a computer-generated random sequence (Research Randomizer Program). The randomization list was prepared and kept by an independent staff member (F.M.) who was not involved in treatment delivery or outcome assessment, ensuring allocation concealment.

Allocation concealment was further achieved using sequentially numbered, opaque, sealed envelopes, which were randomly selected by the participants. The envelope was opened by the operator only after cavity preparation, and the selected tooth was then assigned to either the EQUIA group or BEAUTIFIL group, thereby minimizing performance bias.

**Fig. 1:**
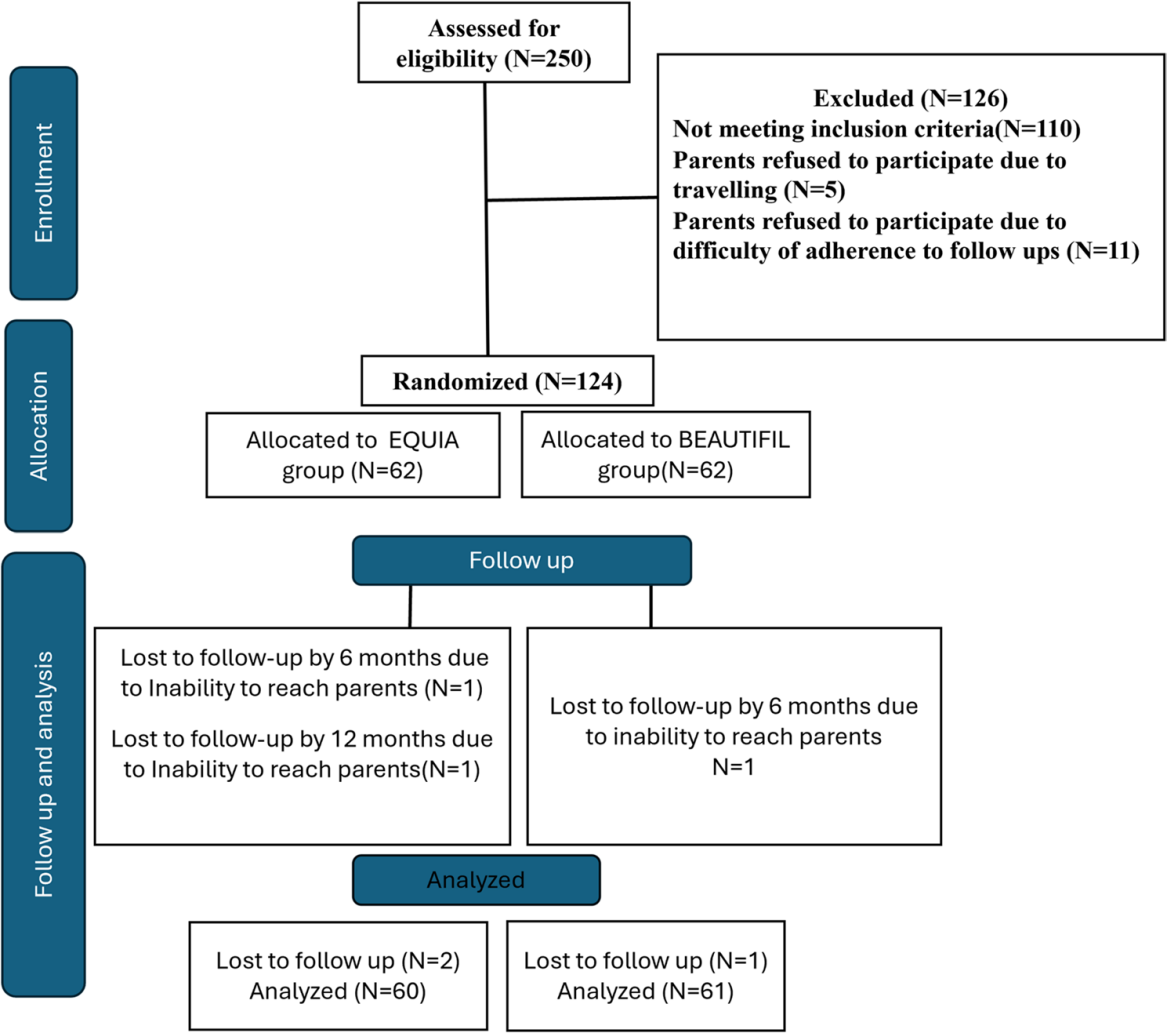
Trial flow chart of study design according to CONSORT

The flow of participants and teeth through the different stages of the trial is illustrated in the CONSORT flowchart Fig. 1.

### Blinding

The participants were blinded to which group they were assigned to. Evaluations were carried out by two well-trained Pediatric dentists (Associate Professors at the Pediatric Dentistry and Dental Public Health Department at Faculty of Dentistry, Ain Shams University), who were not involved in the placement of the restorations and were therefore blinded to the group assignment to avoid assessment bias. On the other hand, the operator/primary investigator was already familiar with the nature of the materials used and their different modes of application, so the operator was not blinded. Blinding of the data analyst was also performed, where all study data were labelled with nonidentifying terms to ensure the objectivity of the study findings and reduce the risk of bias.

### Intervention

After completing both clinical and radiographic assessments, the principal investigator carried out the treatment according to the following protocol.

Local anaesthesia with 4% articaine (Artinibsa, Spain) was used. Class II carious primary molars were then prepared using blue-coded diamond burs (pear-1-8-mm blue-coded diamond-bur OSUNG ASU) at high speed with water cooling to access the carious lesion and remove all peripheral decay. A sharp, dental excavator ZIFFERO (ZFE001, LASCOD) was subsequently used to eliminate soft, infected carious dentin. A conservative cavity design was employed, emphasizing selective caries removal to firm dentin, with no bevelling of cavity walls to preserve as much healthy dental tissue as possible. No additional retention features were created. Finally, the cavity walls were finished using yellow-coded pear-shaped diamond stones (pear-1-8-mm yellow-coded diamond-bur OSUNG ASU). The cavity was cleaned by rubbing wet cotton pellets against its walls to remove debris and remnants of decayed tissue removal. In the presence of the adjacent tooth, saddle matrix (Tor VM, Moscow, Russia) was used and secured in position using Spring-clip (Tor VM, Moscow, Russia) and wooden wedges (Tor VM, Moscow, Russia). The operative field was isolated with the aid of cotton rolls and a saliva ejector.

### Restorative material application

All materials were applied according to manufacturer instructions.

#### EQUIA group

The cavity was conditioned with a 20% poly- acrylic acid ‘dentin conditioner’ (EQUIA GC, JAPAN) for 10 s using micro-applicator (Cotisen, Hebei, China) according to the manufacturer’s instructions. Then, conditioner was rinsed thoroughly for 10 s followed by gentle dryness using mild air blow to avoid desiccation of dentin until the surfaces appeared moist and glistening but not desiccated. Each capsule of highly viscous glass ionomer cement was mixed for 10 s using amalgamator (SDI, Victoria, Australia) according to the manufacturer’s instructions. Immediately, the mixed capsule was removed from the mixer and loaded into the GC Capsule Applier (GC, Tokyo, Japan) then packed into the prepared cavity. Then, restoration surface was countered using a ball burnisher (Critical Dental, Sydney, Australia) and plastic instruments (Critical Dental, Sydney, Australia).Finally, a thin layer of the Equia Forte Coat^®^ (GC Corp) was applied with a micro applicator 150 s after the activation of the EQUIA Forte ^®^ HT Fil capsule and then light-cured for 20 s using a visible light-curing unit with an intensity of 2300 mW/cm², ensuring optimal polymerization of the material. The restoration was evaluated for premature contacts or high spots using articulating paper (ZZlinlker Articulating paper, china). Any identified areas were adjusted and removed with a yellow-coded diamond bur. Finishing was done after 2 min and 30 s using superfine diamond burs under water spray, then reapplication of the EQUIA Forte coat and light curing was done. Finally, the patient was instructed to consume a soft diet and to avoid hard, crunchy, or sticky foods that could dislodge or damage the restoration in the first 48 h post restoration to allow the material to reach its maximum strength and hardness.

#### BEAUTIFIL group

Selective etching of enamel was performed using 37% phosphoric acid gel (Meta Biomed, Korea) which was applied only to the enamel surrounding the margin of prepared cavity and left in place for 60 s. The etched surface was thoroughly rinsed (at least 45 s) with water and then dried with mild air. Drying the cavity was carried using gentle air drying for 4–5 s. In case the cavity was contaminated with blood, saliva or exudates, thorough cleaning of the cavity using 37% phosphoric acid for 2–3 s was done to promote adhesion to tooth structure. A thin layer of the Universal Adhesive BeautiBond (Shofu Inc) was applied using a disposable applicator for at least 10 s and any excess was removed with a bond brush (Cotisen, Hebei, China), then gentle air was blown for 5 s until the runny surface of bond stayed in the same position without movement. Light curing was performed using LED light curing unit (WOODPECKER i-LED Plus, Shanghai Handy Medical Equipment, Shanghai, China) with light intensity of 2300mw/cm2for 10 s, with the light curing tip being placed as close as possible to the surface of the cavity. The Beautifil-Flow Plus X was placed in 2 mm increments creating the desired shape. Each increment was light cured for 20 s at each restoration surface. An articulating paper was used to exclude any occlusal interference. Finishing was done using finishing discs (Tor VM, Moscow, Russia) and / or carbide finishing burs (Kerr, Envista Holdings Corporation, USA). For polishing, Kerr Opti 1 Step Polisher (Kerr, Envista Holdings Corporation, USA) used.

### Clinical assessment

The clinical performance of the two restorative materials was independently assessed by two co-investigators using the FDI evaluation criteria [35] at 3-, 6-, and 12-month recalls following a previously published protocol [36]. The assessment framework included three main domains: esthetic, functional, and biological; comprising fourteen specific sub-criteria relevant to this study: surface luster, surface and marginal staining, color match and translucency, esthetic anatomical form, fracture of the material and retention, marginal adaptation, approximal anatomical form, patient’s perception, hypersensitivity and tooth vitality, recurrence of caries, tooth integrity, periodontal response, adjacent mucosa, and oral and general health. Each sub-criterion was scored on a 5-point scale, where scores of 1–3 were considered clinically acceptable and scores of 4–5 clinically unacceptable. The lowest score within each domain determined the domain outcome, and the lowest domain score was taken as the overall restoration rating. (Additional File 2)

Postoperative hypersensitivity was assessed for each restoration according to the FDI evaluation criteria. Because the study population included children aged 4–8 years, the assessment was adapted to be age-appropriate. Children were asked in simple language whether they experienced discomfort when consuming cold or sweet foods, using examples they could understand (e.g., “Does it hurt when you eat ice cream?“). Parents were also asked to confirm their child’s responses to ensure accuracy. Each response was then assigned a score from 1 to 5 according to the FDI criteria, where 1 indicates no sensitivity and 5 indicates severe discomfort. All assessments were performed by a calibrated examiner to ensure consistency and reliability.

Both examiners performed independent, single evaluations of all restorations.

In instances where inconsistencies were identified between their assessments, a consensus was reached; if disagreement persisted, the lower score was recorded.

To ensure the reliability and reproducibility of the clinical assessments, both inter-examiner and intra-examiner reliability were evaluated. Intra-examiner reliability was assessed by having the same examiner evaluate four restorations on two separate occasions, with a one-week interval between assessments, under similar clinical conditions. Inter-examiner reliability was evaluated by having the two independent, calibrated examiners assess twelve molars: six restored with BEAUTIFIL Flow Plus X and six with EQUIA Forte HT Fil using the FDI evaluation criteria.

These pilot assessments were conducted prior to the conduction of the study to verify calibration consistency and were therefore excluded from the final statistical analysis.

Before initiating the main evaluation phase, both examiners participated in calibration sessions designed to standardize the interpretation and application of the FDI scoring system.

Statistical analysis of examiner agreement using Cohen’s kappa coefficient demonstrated excellent reliability, with values of 0.89 for intra-examiner and 0.85 for inter-examiner consistency.

### Statistical analysis

Numerical data were presented as mean, standard deviation (SD), median, and interquartile range values. They were tested for normality using Shapiro-Wilk’s test. Age data were found to be normally distributed and were compared using an independent t-test. Ordinal and categorical data were presented as frequency and percentage values. Categorical data were analyzed using the chi-square test. Ordinal and non-parametric numerical data were analyzed using Mann-Whitney and signed rank tests for intergroup and intragroup comparisons, respectively. P-values were adjusted for multiple comparisons using the False Discovery Rate method. The significance level was set at *p* < 0.05 within all tests. Statistical analysis was performed with R statistical analysis software version 4.4.2 for Windows.

## Results

### Handling of missing data

In the present study, the patients who dropped out during the follow-up were contacted by phone. There were no complaints about the restoration, and the restoration was intact; however, they did not show up because of distant residence. Therefore, the missing data was considered to be “missing completely at random” data (MCAR), i.e., the probability of data being missing was the same for all observations. Out of the 124 primary molars in 124 patients allocated in this study, two patients were lost throughout the follow-up period in the EQUIA group, and one patient in the BEAUTIFIL group. One patient dropped out in the six-month follow-up, and the other dropped out in the 12-month follow-up in the EQUIA group, while in the BEAUTIFIL group, one patient dropped out in the six-month follow-up period despite several scheduled appointments. Those 3 dropouts were excluded from the study as they were in the specified percentage of 15% dropouts. The analysis was performed on only 60 molars in the EQUIA group and 61 molars in the BEAUTIFIL group.

### Demographic data

Intergroup and summary statistics for demographic data are presented in Table 1.

**Table 1:** Intergroup and summary statistics for demographic data

Parameter		EQUIA Group	BEAUTIFIl group	*p*-value
Sex [n (%)]	**Male**	36 (60.00%)	32 (52.46%)	0.465ns
	**Female**	24 (40.00%)	29 (47.54%)	
Age (years)	**Mean ± SD**	6.05 ± 1.08	6.08 ± 1.02	0.491ns
	**Median (IQR)**	6.00 (2.00)	6.00 (2.00)	
Treated arch [n (%)]	**Upper**	33 (55.00%)	28 (45.90%)	0.365ns
	**Lower**	27 (45.00%)	33 (54.10%)	
Treated tooth [n (%)]	**First molar**	25 (41.67%)	24 (39.34%)	0.854ns
	**Second molar**	35 (58.33%)	37 (60.66%)	
Cavity location [n (%)]	**Mesial**	36 (60.00%)	38 (62.30%)	0.853ns
	**Distal**	24 (40.00%)	23 (37.70%)	
DMF	**Mean ± SD**	1.17 ± 0.41	3.00 ± 1.15	0.015*
	**Median (IQR)**	1.00 (0.00)	3.00 (2.00)	
Def	**Mean ± SD**	6.58 ± 2.06	6.07 ± 2.33	0.485ns
	**Median (IQR)**	7.00 (2.50)	6.00 (3.00)	
Dmf	**Mean ± SD**	7.55 ± 2.53	7.45 ± 2.32	0.907ns
	**Median (IQR)**	7.00 (4.00)	8.00 (2.00)	
Caries experience [n (%)]	**Low** $\:\le\:$ **3**	3 (5.00%)	4 (6.56%)	0.853ns
	**High > 3**	57 (95.00%)	57 (93.44%)	

*ns* not significant

The study analysis was done on 121 cases, with 60 cases allocated to the EQUIA group and 61 to the BEAUTIFIL group.

### Esthetic properties

Table 2 shows the esthetic properties of both restorations at different follow-up periods.

**Table 2:** Inter and intragroup comparisons of scores of different esthetic properties

Interval	Score	Surface luster [*n* (%)]		*p*-value	Staining [*n* (%)]		*p*-value	Color match & Translucency [*n* (%)]		*p*-value	Esthetic Anatomical Form [*n* (%)]		*p*-value
		EQUIA Forte HT Fil	BEAUTIFIl Flow Plus X		EQUIA Forte HT Fil	BEAUTIFIl Flow Plus X		EQUIA Forte HT Fil	BEAUTIFIl Flow Plus X		EQUIA Forte HT Fil	BEAUTIFIl Flow Plus X	
3 months	**1**	40 (66.67%)^B^	60 (98.36%)^B^	< 0.001*	55 (91.67%)^B^	60 (98.36%)^B^	0.248ns	37 (61.67%)^B^	58 (95.08%)^B^	<0.001*	41 (68.33%)^B^	55 (90.16%)^B^	0.006*
	**2**	11 (18.33%)	0 (0.00%)		2 (3.33%)	1 (1.64%)		17 (28.33%)	2 (3.28%)		13 (21.67%)	6 (9.84%)	
	**3**	7 (11.67%)	1 (1.64%)		1 (1.67%)	0 (0.00%)		4 (6.67%)	1 (1.64%)		4 (6.67%)	0 (0.00%)	
	**4**	0 (0.00%)	0 (0.00%)		0 (0.00%)	0 (0.00%)		0 (0.00%)	0 (0.00%)		0 (0.00%)	0 (0.00%)	
	**5**	2 (3.33%)	0 (0.00%)		2 (3.33%)	0 (0.00%)		2 (3.33%)	0 (0.00%)		2 (3.33%)	0 (0.00%)	
6 months	**1**	38 (63.33%)^A^	54 (88.52%)^A^	0.002*	53 (88.33%)^A^	56 (91.80%)^A^	0.478ns	35 (58.33%)^B^	52 (85.25%)^A^	< 0.001*	38 (63.33%)^A^	45 (73.77%)^A^	0.210ns
	**2**	13 (21.67%)	4 (6.56%)		2 (3.33%)	4 (6.56%)		19 (31.67%)	8 (13.11%)		14 (23.33%)	14 (22.95%)	
	**3**	6 (10.00%)	2 (3.28%)		2 (3.33%)	0 (0.00%)		3 (5.00%)	0 (0.00%)		4 (6.67%)	1 (1.64%)	
	**4**	0 (0.00%)	0 (0.00%)		0 (0.00%)	0 (0.00%)		0 (0.00%)	0 (0.00%)		1 (1.67%)	0 (0.00%)	
	**5**	3 (5.00%)	1 (1.64%)		3 (5.00%)	1 (1.64%)		3 (5.00%)	1 (1.64%)		3 (5.00%)	1 (1.64%)	
12 months	**1**	34 (56.67%)^A^	51 (83.61%)^A^	0.002*	48 (80.00%)^A^	54 (88.52%)^A^	0.248ns	29 (48.33%)^A^	49 (80.33%)^A^	< 0.001*	37 (61.67%)^A^	42 (68.85%)^A^	0.306ns
	**2**	14 (23.33%)	5 (8.20%)		4 (6.67%)	5 (8.20%)		22 (36.67%)	9 (14.75%)		15 (25.00%)	15 (24.59%)	
	**3**	6 (10.00%)	3 (4.92%)		2 (3.33%)	0 (0.00%)		3 (5.00%)	1 (1.64%)		1 (1.67%)	2 (3.28%)	
	**4**	0 (0.00%)	0 (0.00%)		0 (0.00%)	0 (0.00%)		0 (0.00%)	0 (0.00%)		1 (1.67%)	0 (0.00%)	
	**5**	6 (10.00%)	2 (3.28%)		6 (10.00%)	2 (3.28%)		6 (10.00%)	2 (3.28%)		6 (10.00%)	2 (3.28%)	
*p*-value		0.003*	< 0.001*		0.005*	0.005*		< 0.001*	0.006*		0.013*	< 0.001*	

Values with different superscripts within the same vertical column are significantly different, * significant (*p* < 0.05), ns not significant

For surface luster and color match measured in different intervals, and for esthetic anatomical form measured after 3 months, EQUIA group had significantly higher scores than BEAUTIFIl group. However, for other parameters, the differences were not statistically significant, with the majority of all cases having a score of (1).

For all esthetic properties, there was a significant increase in measured scores in different intervals within both groups. For color match in EQUIA group, the increase was statistically significant after 12 months. However, for other groups, the increase started after 6 months.

Within all intervals, there was a significant difference in the total score, with EQUIA group having significantly higher scores than the BEAUTIFIl group. Table 3 shows the total esthetic scores of both restorations at the different follow-up periods.

**Table 3:** Inter and intragroup comparisons of scores of different esthetic properties

Interval	Total esthetic Score	[*n* (%)]		*p*-value
		EQUIA Forte HT Fil	BEAUTIFIl Flow Plus X	
3 months	**1**	31 (51.67%)^B^	54 (88.52%)^B^	< 0.001*
	**2**	18 (30.00%)	6 (9.84%)	
	**3**	9 (15.00%)	1 (1.64%)	
	**4**	0 (0.00%)	0 (0.00%)	
	**5**	2 (3.33%)	0 (0.00%)	
6 months	**1**	28 (46.67%)^B^	44 (72.13%)^A^	< 0.001*
	**2**	19 (31.67%)	13 (21.31%)	
	**3**	9 (15.00%)	3 (4.92%)	
	**4**	1 (1.67%)	0 (0.00%)	
	**5**	3 (5.00%)	1 (1.64%)	
12 months	**1**	22 (36.67%)^A^	41 (67.21%)^A^	< 0.001*
	**2**	25 (41.67%)	14 (22.95%)	
	**3**	6 (10.00%)	4 (6.56%)	
	**4**	1 (1.67%)	0 (0.00%)	
	**5**	6 (10.00%)	2 (3.28%)	
*p*-value		< 0.001*	< 0.001*	

Values with different superscripts within the same vertical column are significantly different, * significant (*p* < 0.05), ns not significant

### Functional properties

Inter and intragroup comparisons of scores of different functional properties are presented in Table 4.

**Table 4:** Inter and intragroup comparisons of scores of different functional properties

Interval	Score	Fracture of material and retention [*n* (%)]		*p*-value	Marginal adaptation [*n* (%)]		*p*-value	Approximal anatomical form [*n* (%)]		*p*-value	Patients view [*n* (%)]		*p*-value
		EQUIA Forte HT Fil	BEAUTIFIl Flow Plus X		EQUIA Forte HT Fil	BEAUTIFIl Flow Plus X		EQUIA Forte HT Fil	BEAUTIFIl Flow Plus X		EQUIA Forte HT Fil	BEAUTIFIl Flow Plus X	
3 months	**1**	54 (90.00%)^B^	61 (100.00%)^B^	0.036*	50 (83.33%)^A^	59 (96.72%)^C^	0.039*	52 (86.67%)^B^	58 (95.08%)^B^	0.097ns	52 (86.67%)^B^	60 (98.36%)^B^	0.042*
	**2**	3 (5.00%)	0 (0.00%)		7 (11.67%)	2 (3.28%)		4 (6.67%)	3 (4.92%)		6 (10.00%)	1 (1.64%)	
	**3**	1 (1.67%)	0 (0.00%)		1 (1.67%)	0 (0.00%)		2 (3.33%)	0 (0.00%)		0 (0.00%)	0 (0.00%)	
	**4**	0 (0.00%)	0 (0.00%)		0 (0.00%)	0 (0.00%)		0 (0.00%)	0 (0.00%)		0 (0.00%)	0 (0.00%)	
	**5**	2 (3.33%)	0 (0.00%)		2 (3.33%)	0 (0.00%)		2 (3.33%)	0 (0.00%)		2 (3.33%)	0 (0.00%)	
6 months	**1**	52 (86.67%)^A^	58 (95.08%)^A^	0.116ns	48 (80.00%)^A^	53 (86.89%)^B^	0.399ns	46 (76.67%)^A^	54 (88.52%)^A^	0.097ns	51 (85.00%)^A^	56 (91.80%)^A^	0.352ns
	**2**	3 (5.00%)	0 (0.00%)		7 (11.67%)	7 (11.48%)		4 (6.67%)	5 (8.20%)		6 (10.00%)	4 (6.56%)	
	**3**	1 (1.67%)	1 (1.64%)		1 (1.67%)	0 (0.00%)		7 (11.67%)	1 (1.64%)		0 (0.00%)	0 (0.00%)	
	**4**	1 (1.67%)	1 (1.64%)		1 (1.67%)	0 (0.00%)		0 (0.00%)	0 (0.00%)		0 (0.00%)	0 (0.00%)	
	**5**	3 (5.00%)	1 (1.64%)		3 (5.00%)	1 (1.64%)		3 (5.00%)	1 (1.64%)		3 (5.00%)	1 (1.64%)	
12 months	**1**	48 (80.00%)^A^	56 (91.80%)^A^	0.099ns	47 (78.33%)^A^	47 (77.05%)^A^	0.957ns	45 (75.00%)^A^	53 (86.89%)^B^	0.097ns	49 (81.67%)^A^	53 (86.89%)^A^	0.376ns
	**2**	5 (8.33%)	1 (1.64%)		6 (10.00%)	11 (18.03%)		3 (5.00%)	5 (8.20%)		5 (8.33%)	6 (9.84%)	
	**3**	0 (0.00%)	0 (0.00%)		0 (0.00%)	0 (0.00%)		6 (10.00%)	1 (1.64%)		0 (0.00%)	0 (0.00%)	
	**4**	1 (1.67%)	2 (3.28%)		1 (1.67%)	1 (1.64%)		0 (0.00%)	0 (0.00%)		0 (0.00%)	0 (0.00%)	
	**5**	6 (10.00%)	2 (3.28%)		6 (10.00%)	2 (3.28%)		6 (10.00%)	2 (3.28%)		6 (10.00%)	2 (3.28%)	
*p*-value		0.008*	0.018*		0.135ns	< 0.001*		< 0.001*	0.015*		0.022*	0.010*	

Values with different superscripts within the same vertical column are significantly different, * significant (*p* < 0.05), ns not significant

For fracture & retention, marginal adaptation, and patients’ view measured after 3 months, the EQUIA group had significantly higher scores than BEAUTIFIl group. However, for other parameters and intervals, the differences were not statistically significant. Table 5 shows that within all intervals, there was no significant difference in the total score measured in both groups, with the majority of cases having a score of (1). Within both groups, there was a significant difference between scores measured in different intervals, with a significant increase in observed scores after 6 months.

**Table 5:** Inter and intragroup comparisons of scores of different functional properties

Interval	Total functional Score	[*n* (%)]		*p*-value
		EQUIA Forte HT Fil	BEAUTIFIl Flow Plus X	
3 months	**1**	47 (78.33%)^B^	56 (91.80%)^B^	0.087ns
	**2**	7 (11.67%)	5 (8.20%)	
	**3**	4 (6.67%)	0 (0.00%)	
	**4**	0 (0.00%)	0 (0.00%)	
	**5**	2 (3.33%)	0 (0.00%)	
6 months	**1**	43 (71.67%)^A^	48 (78.69%)^A^	0.323ns
	**2**	6 (10.00%)	10 (16.39%)	
	**3**	7 (11.67%)	1 (1.64%)	
	**4**	1 (1.67%)	1 (1.64%)	
	**5**	3 (5.00%)	1 (1.64%)	
12 months	**1**	41 (68.33%)^A^	45 (73.77%)^A^	0.323ns
	**2**	7 (11.67%)	12 (19.67%)	
	**3**	5 (8.33%)	0 (0.00%)	
	**4**	1 (1.67%)	2 (3.28%)	
	**5**	6 (10.00%)	2 (3.28%)	
*p*-value		0.003*	< 0.001*	

Values with different superscripts within the same vertical column are significantly different, * significant (*p* < 0.05), ns not significant

### Biological properties

Inter and intragroup comparisons of scores of different biological properties are presented in Tables 6 and 7.

**Table 6:** Inter and intragroup comparisons of scores of different biological properties (A)

Interval	Score	Hypersensitivity and tooth vitality [*n* (%)]		*p*-value	Recurrence of caries [*n* (%)]		*p*-value	Tooth integrity [*n* (%)]		*p*-value
		EQUIA Forte HT Fil	BEAUTIFIl Flow Plus X		EQUIA Forte HT Fil	BEAUTIFIl Flow Plus X		EQUIA Forte HT Fil	BEAUTIFIl Flow Plus X	
3 months	**1**	58 (96.67%)^A^	61 (100.00%)^A^	0.234ns	58 (96.67%)^A^	61 (100.00%)^A^	0.234ns	58 (96.67%)^A^	61 (100.00%)^A^	0.234ns
	**2**	0 (0.00%)	0 (0.00%)		0 (0.00%)	0 (0.00%)		0 (0.00%)	0 (0.00%)	
	**3**	0 (0.00%)	0 (0.00%)		0 (0.00%)	0 (0.00%)		0 (0.00%)	0 (0.00%)	
	**4**	0 (0.00%)	0 (0.00%)		0 (0.00%)	0 (0.00%)		0 (0.00%)	0 (0.00%)	
	**5**	2 (3.33%)	0 (0.00%)		2 (3.33%)	0 (0.00%)		2 (3.33%)	0 (0.00%)	
6 months	**1**	57 (95.00%)^A^	60 (98.36%)^A^	0.307ns	57 (95.00%)^A^	60 (98.36%)^A^	0.307ns	57 (95.00%)^A^	60 (98.36%)^A^	0.307ns
	**2**	0 (0.00%)	0 (0.00%)		0 (0.00%)	0 (0.00%)		0 (0.00%)	0 (0.00%)	
	**3**	0 (0.00%)	0 (0.00%)		0 (0.00%)	0 (0.00%)		0 (0.00%)	0 (0.00%)	
	**4**	0 (0.00%)	0 (0.00%)		0 (0.00%)	0 (0.00%)		0 (0.00%)	0 (0.00%)	
	**5**	3 (5.00%)	1 (1.64%)		3 (5.00%)	1 (1.64%)		3 (5.00%)	1 (1.64%)	
12 months	**1**	54 (90.00%)^A^	59 (96.72%)^A^	0.234ns	54 (90.00%)^A^	59 (96.72%)^A^	0.234ns	54 (90.00%)^A^	59 (96.72%)^A^	0.234ns
	**2**	0 (0.00%)	0 (0.00%)		0 (0.00%)	0 (0.00%)		0 (0.00%)	0 (0.00%)	
	**3**	0 (0.00%)	0 (0.00%)		0 (0.00%)	0 (0.00%)		0 (0.00%)	0 (0.00%)	
	**4**	0 (0.00%)	0 (0.00%)		0 (0.00%)	0 (0.00%)		0 (0.00%)	0 (0.00%)	
	**5**	6 (10.00%)	2 (3.28%)		6 (10.00%)	2 (3.28%)		6 (10.00%)	2 (3.28%)	
*p*-value		0.078ns	0.223ns		0.078ns	0.223ns		0.078ns	0.223ns	

Values with different superscripts within the same vertical column are significantly different, ns not significant

**Table 7:** Inter and intragroup comparisons of scores of different biological properties (B)

Interval	Score	Periodontal response [*n* (%)]		*p*-value	Adjacent mucosa [*n* (%)]		*p*-value	Oral and general health [*n* (%)]		*p*-value
		EQUIA Forte HT Fil	BEAUTIFIl Flow Plus X		EQUIA Forte HT Fil	BEAUTIFIl Flow Plus X		EQUIA Forte HT Fil	BEAUTIFIl Flow Plus X	
3 months	**1**	56 (93.33%)^A^	60 (98.36%)^A^	0.284ns	56 (93.33%)^A^	60 (98.36%)^A^	0.308ns	57 (95.00%)^A^	61 (100.00%)^A^	0.174ns
	**2**	2 (3.33%)	1 (1.64%)		2 (3.33%)	1 (1.64%)		1 (1.67%)	0 (0.00%)	
	**3**	0 (0.00%)	0 (0.00%)		0 (0.00%)	0 (0.00%)		0 (0.00%)	0 (0.00%)	
	**4**	0 (0.00%)	0 (0.00%)		0 (0.00%)	0 (0.00%)		0 (0.00%)	0 (0.00%)	
	**5**	2 (3.33%)	0 (0.00%)		2 (3.33%)	0 (0.00%)		2 (3.33%)	0 (0.00%)	
6 months	**1**	54 (90.00%)^A^	58 (95.08%)^A^	0.284ns	55 (91.67%)A	59 (96.72%)^A^	0.308ns	56 (93.33%)^A^	60 (98.36%)^A^	0.174ns
	**2**	3 (5.00%)	2 (3.28%)		2 (3.33%)	1 (1.64%)		1 (1.67%)	0 (0.00%)	
	**3**	0 (0.00%)	0 (0.00%)		0 (0.00%)	0 (0.00%)		0 (0.00%)	0 (0.00%)	
	**4**	0 (0.00%)	0 (0.00%)		0 (0.00%)	0 (0.00%)		0 (0.00%)	0 (0.00%)	
	**5**	3 (5.00%)	1 (1.64%)		3 (5.00%)	1 (1.64%)		3 (5.00%)	1 (1.64%)	
12 months	**1**	52 (86.67%)^A^	57 (93.44%)^A^	0.284ns	53 (88.33%)^A^	57 (93.44%)^A^	0.308ns	53 (88.33%)^A^	58 (95.08%)^A^	0.174ns
	**2**	2 (3.33%)	2 (3.28%)		1 (1.67%)	2 (3.28%)		1 (1.67%)	1 (1.64%)	
	**3**	0 (0.00%)	0 (0.00%)		0 (0.00%)	0 (0.00%)		0 (0.00%)	0 (0.00%)	
	**4**	0 (0.00%)	0 (0.00%)		0 (0.00%)	0 (0.00%)		0 (0.00%)	0 (0.00%)	
	**5**	6 (10.00%)	2 (3.28%)		6 (10.00%)	2 (3.28%)		6 (10.00%)	2 (3.28%)	
*p*-value		0.180ns	0.180ns		0.311ns	0.311ns		0.078ns	0.097ns	

Values with different superscripts within the same vertical column are significantly different, ns not significant

For all biological properties measured at different intervals, the majority of cases in both groups had a score of (1), and the difference was not statistically significant.

For all parameters estimated in both groups, there was no significant difference between values in different intervals.

Within all intervals, there was no significant difference in the total score measured in both groups, with the majority of cases having a score of (1). Table 8.

**Table 8:** Inter and intragroup comparisons of scores of different biological properties

Interval	Total biological Score	[*n* (%)]		*p*-value
		EQUIA Forte HT Fil	BEAUTIFIl Flow Plus X	
3 months	**1**	56 (93.33%)^A^	60 (98.36%)^A^	0.284ns
	**2**	2 (3.33%)	1 (1.64%)	
	**3**	0 (0.00%)	0 (0.00%)	
	**4**	0 (0.00%)	0 (0.00%)	
	**5**	2 (3.33%)	0 (0.00%)	
6 months	**1**	54 (90.00%)^A^	58 (95.08%)^A^	0.284ns
	**2**	3 (5.00%)	2 (3.28%)	
	**3**	0 (0.00%)	0 (0.00%)	
	**4**	0 (0.00%)	0 (0.00%)	
	**5**	3 (5.00%)	1 (1.64%)	
12 months	**1**	52 (86.67%)^A^	57 (93.44%)^A^	0.284ns
	**2**	2 (3.33%)	2 (3.28%)	
	**3**	0 (0.00%)	0 (0.00%)	
	**4**	0 (0.00%)	0 (0.00%)	
	**5**	6 (10.00%)	2 (3.28%)	
*p*-value		0.180ns	0.180ns	

Values with different superscripts within the same vertical column are significantly different, * significant (*p* < 0.05), ns not significant

### Success rate

**Fig. 2:**
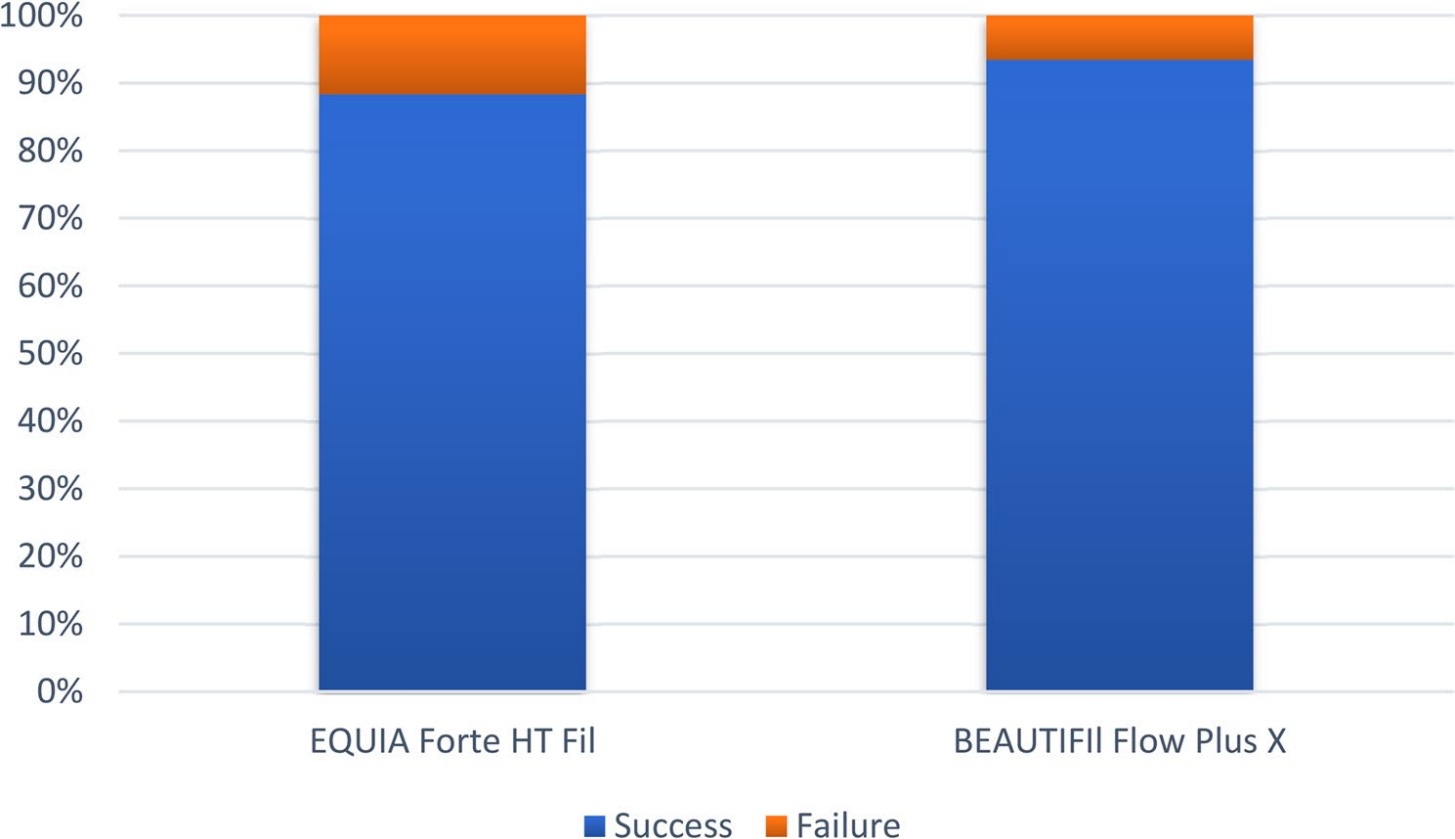
Stacked bar chart for overall success rate

Intergroup comparison of clinical outcomes is presented in Table 9, and Fig. 2.

**Table 9:** Intergroup comparison of overall clinical outcome

Clinical outcome	[*n* (%)]		*p*-value
	EQUIA Forte HT Fil	BEAUTIFIl Flow Plus X	
Success	53 (88.33%)	57 (93.44%)	0.363ns
Failure	7 (11.67%)	4 (6.56%)	

*ns* not significant

There were 7 failed cases in the EQUIA group and 4 in the BEAUTIFIl group, and the difference was not statistically significant.

## Discussion

Effective restoration of primary teeth is dependent on several factors, i.e., the compliance of the child, operator skills in both behavioral and technical management, and finally, the individual properties of the chosen restorative biomaterial [37]. Proximal cavities in primary molars pose additional challenges owing to their anatomical characteristics. The reduced enamel and dentin thickness often results in less retentive cavities, which may increase the risk of restoration failure. Considering these challenges, no single restorative material has yet proven ideal for all primary teeth restorations [38]. Thus, the search for adhesive restorative materials to improve the success rates of proximal restorations is still necessary.

While glass ionomers with hybrid technology have demonstrated promising results in terms of handling characteristics and initial clinical performance [39], the available data on their long-term clinical efficacy, particularly in Class II restorations of primary teeth, are relatively sparse. Moreover, the literature presents conflicting findings regarding performance and durability [40, 41] .Similarly, although giomer restorative materials have been available for some years and present good behavior and good mechanical stability [42], the clinical evidence that supports their use is still scarce, again related to primary teeth. These gaps underscore the need for well-designed clinical trials to evaluate their reliability and durability in pediatric restorative dentistry.

In terms of esthetic outcomes, the BEAUTIFIL group demonstrated superior surface luster and color match compared to the EQUIA group. Surface luster, reflecting the smoothness and light-reflecting ability of a restoration, is a key determinant of esthetic success and is inversely related to surface roughness [43]. The enhanced esthetic performance of the BEAUTIFIL group may be attributed to its resin-containing structure, which reduces surface roughness and produces a smoother finish. A smoother surface not only enhances light reflection and esthetic appeal but may also facilitate oral hygiene and reduce plaque accumulation, contributing to the long-term maintenance of restoration appearance. These findings are consistent with previous studies reporting that glass hybrid materials tend to exhibit greater surface roughness compared to resin-based restoratives, which can affect their clinical appearance [44, 45].

Within the EQUIA group, surface luster showed a gradual decrease over time, consistent with previous studies (Eissa et al., [46] Kamal et al., [47] Fatma et al. [48]). This decline may be related to the wear of the protective surface coating during mastication, highlighting the importance of periodic replacement to prevent clinical and long-term complications (Balkaya et al. [49]). In the BEAUTIFIL group, the minor reduction in surface luster overtime could be attributed to the hybrid composition, where filler particle loss and matrix degradation under oral conditions may slightly roughen the surface [50]. These findings emphasize the need to consider material composition and long-term stability when selecting restoratives for primary molars.

Regarding marginal and surface staining, no significant differences were observed between the groups, with minimal staining that was easily removed and clinically insignificant. The slightly better performance of the BEAUTIFIL group may be attributed to its resin-based composition and S-PRG fillers, which mimic the optical properties of natural teeth. These findings are consistent with previous reports indicating that hybrid materials like BEAUTIFIL II are less prone to staining [51, 52]. However, Ozer et al. [26] observed significant discoloration over 36 months, indicating the need for longer-term studies to fully assess color stability. The absence of marginal discoloration also reflects good bonding of both materials to the tooth structure without microleakage, supporting their clinical reliability in proximal restorations of primary molars.

Concerning color match and translucency, the BEAUTIFIL group demonstrated superior outcomes compared to the EQUIA group. This advantage may be attributed to its advanced resin matrix and highly polishable surface. The filler structure has been reported to closely mimic the internal morphology of natural dental tissues, providing ideal translucency and optical behavior comparable to sound tooth structure. Additionally, the material exhibits a chameleon effect, allowing a single shade to blend with surrounding teeth, making the restoration nearly undetectable. These properties collectively enable BEAUTIFIL Flow Plus X to closely mimic the natural appearance of teeth, contributing to improved esthetic outcomes [53].​.

With respect to the esthetic anatomical form, no notable differences were observed between the two groups after 12 months of follow-up, with both restorations showing comparable resistance to anatomical changes. This outcome may be attributed to the application of a resin coating on Equia Forte restorations, which has been reported to enhance the mechanical properties, including strength and abrasion resistance, of glass hybrid materials [54].

Overall, the BEAUTIFIL group demonstrated superior esthetic scores compared to the EQUIA group, although both restorations were considered clinically acceptable at all evaluation intervals according to FDI criteria. Patients generally accepted the esthetics of both types of restorations, indicating that despite the measurable differences, both materials provide satisfactory outcomes from a patient perspective.

Although clinicians detected statistically significant differences in esthetic parameters, patients generally reported no difference in the acceptance of the two restorative materials. This discrepancy underscores an important distinction between professional esthetic evaluation and patient-perceived clinical relevance. Clinicians are trained to identify subtle differences in surface characteristics such as luster and texture using standardized criteria like the FDI system; however, these differences may fall below the threshold of perception for patients or parents. Clinically, this suggests that statistically significant esthetic differences do not necessarily influence patient satisfaction or acceptance. This distinction highlights that statistical significance does not necessarily imply clinical relevance, particularly for esthetic parameters that involve subtle surface characteristics. Therefore, both materials can be considered esthetically acceptable from the patient’s viewpoint, despite measurable differences identified during professional assessment.

Regarding the functional properties, after 12 months, both the EQUIA and BEAUTIFIL groups demonstrated comparable functional performance, with no notable differences in material fracture. These findings are consistent with Nassar et al. [55], who reported no significant differences in fracture and retention among different bioactive restorative materials (Activa bioactive composite, Beautifil II, and Fuji IX GP EXTRA). Additionally, a previous in vitro study reported that Equia Forte and composite resin materials showed similar flexural strength and hardness values [39]. While some literature suggests that glass ionomers are more prone to fracture compared to resin composites, the improved performance of Equia Forte in this study could be attributed to its enhanced fracture toughness and durability. The application of protective surface coating further contributes to mechanical strength by preventing contamination from intraoral fluids prior to hardening, which can otherwise compromise the material’s matrix and reinforcing properties [43].

The high retention performance observed for BEAUTIFIL restorations in the present study is consistent with previous clinical investigations reporting excellent long-term stability of these materials. Similar high retention outcomes have been documented by Nassar et al. [55], and Samir et al. [52], supporting the reliability of giomer restorations in pediatric proximal cavities. Variations in reported retention rates across studies may be related to differences in cavity location, occlusal load, and clinical conditions. In contrast, lower long-term retention has been reported in studies with extended follow-up periods, such as that of Gordan et al. [56] who observed a decline in retention over 13 years, highlighting the influence of time on restorative survival.

The retention performance of EQUIA Forte observed in this study also aligns with previous findings. Atmaca et al. [57], reported comparable success rates for EQUIA Forte in Class II cavities, suggesting that improvements in glass hybrid formulations and the use of surface coating may enhance retention and fracture resistance, making these materials clinically acceptable for proximal restorations in primary teeth.

As per marginal adaptation, both restorative materials demonstrated favorable performance over the one-year follow-up period, with only rare instances of minor marginal defects requiring repair with no statistically significant differences between the groups. The limited incidence of marginal discrepancies observed in both groups suggests that both materials are capable of maintaining clinically acceptable marginal integrity in Class II restorations of primary teeth. The comparable marginal adaptation of both groups came in an agreement with the findings of Nassar et al. [55] and Kharma et al. [58] reporting no significant difference in the marginal adaptation between EQUIA and other tested materials by the end of 1-year follow up period.

The satisfactory marginal adaptation of glass ionomer restorations may be attributed to the presence of a protective resin coating, which seals the restoration margins and shields them from porosities, cracks, and external contamination. In addition, the chemical adhesion of glass ionomer cement to tooth structure contributes to the formation of an acid- and base-resistant interfacial layer, resulting in a durable marginal seal. The dimensional stability of glass ionomer cement (GIC) can be attributed to its hydrophilic nature, which allows absorption of moisture from the prepared cavity, while its chemical adhesion to enamel contributes to maintaining an effective marginal seal. Additionally, GIC has the same coefficient of thermal expansion as the tooth structure, and the micro gap between the restoration and a tooth is small [43].Collectively, these material-related properties may explain the favorable marginal adaptation observed in the present study and support the clinical reliability of glass ionomer-based restorations in primary molars.

The favorable marginal adaptation and minimal marginal staining observed in the BEAUTIFIL group may be attributed to the improved adaptation and void-free placement associated with injectable restorative materials. The low viscosity and enhanced flowability of giomer-based injectable composites allow intimate adaptation to cavity walls, potentially reducing interfacial gaps. In addition, their elastic properties may enable stress absorption and redistribution at the tooth–restoration interface, thereby limiting marginal deterioration. These findings are consistent with those of Hendam et al. [59], who reported that giomer-based injectable composite has lower viscosity, increased flowability, and elasticity, justifying improved marginal adaptation and reduced marginal discoloration.

Regarding proximal contact integrity, no statistically significant differences were observed between the EQUIA and BEAUTIFIL groups at any evaluation interval, which may be attributed to the use of a standardized and reliable matricing technique in both groups. This finding highlights the critical role of operative technique, independent of the restorative material, in achieving adequate proximal contact.

However, within-group analysis revealed a significant deterioration in proximal contact scores over time in the EQUIA group, particularly at the 12-month evaluation compared to baseline. This intragroup change suggests gradual material loss rather than an immediate failure of proximal contact formation. Similar findings by Scholtanus & Huysmans [60], who reported material loss on the proximal surfaces of the restorations, as observed on the radiographs, usually below the contact sites. Moreover, Klinke et al. [61], Tal et al. [62] Balkaya et al. [63], and Eissa et al. [46] attributed interproximal material loss to the inability of the protective coating to cover the proximal surface of the restorations, which causes this surface to be unprotected from moisture contamination during the maturation phase. Minor time-dependent changes were also observed within the BEAUTIFIL (giomer) group. These are likely related to gradual wear of the resin matrix or filler particle adjustments under functional loading, reflecting material-specific adaptation rather than clinically relevant deterioration. Overall, both materials maintained satisfactory proximal contact over the one-year follow-up period.

Overall, no significant difference was observed in the functional performance between the EQUIA and BEAUTIFIL (giomer) groups throughout the follow-up period. Both materials demonstrated satisfactory fracture resistance, retention, and marginal adaptation, indicating comparable clinical reliability in restoring Class II cavities in primary molars. Minor time-dependent changes within each group reflect normal material-specific adaptation under functional stress, without compromising the overall functional outcome.

For all the biological properties measured at different intervals, the majority of the cases in both groups had a score of (1), and the difference was not statistically significant. Additionally, for all parameters estimated in both groups, there was no significant difference between values at different intervals, suggesting that both materials perform equally well in maintaining tooth vitality, preventing caries, preserving tooth integrity and having similar impacts on the periodontal response, adjacent mucosa, and oral/general health.

Throughout the study, no recurrent caries was observed, indicating that both materials effectively sealed cavities and prevented bacterial infiltration. This is aligned with studies conducted by Eissa et al. [46] Samir et al. [52], which demonstrated that fluoride releasing, and bioactive materials provide significant caries protection. However, longer term studies are needed to assess the sustained anti-cariogenic effects. This positive result could be attributed to several factors. First, the relatively short follow-up period may have limited the time for secondary caries to develop and become noticeable. It is also important to consider participants’ oral hygiene habits and individual caries risk, as these factors play a significant role in the development of secondary caries. In this study, great emphasis was placed on promoting proper oral and dental hygiene practices among the participants.

Moreover, the bioactive flowable giomer material used has antibacterial properties that can hinder the activity of cariogenic bacteria such as Streptococcus mutans. These compounds contribute to the formation of fluorapatite, which is more acid resistant than hydroxyapatite is, along with the ability to remineralize initial carious lesions. Ozer et al. [26] reported that the S-PRG filler particles in giomers act as a fluoride reservoir that recharges with rinsing or brushing with fluoridated products. Giomers form an acid-resistant film that resists the formation of plague, as it hinders bacterial adherence, all of which may have contributed to the prevention of secondary caries.

At the 12-month follow-up, both the EQUIA and BEAUTIFIL groups demonstrated high clinical success, with no significant differences observed between them. The success rate of Equia Forte restorations in our study was comparable to previous reports, such as Gurgan et al. [64] (93.7% at 12 months) and Atmaca et al. [57] (90% in Class II cavities), confirming the material’s reliable performance. The BEAUTIFIL group showed a 12-month success rate of 93.44%, consistent with Sengul and Gurbuz [65] (89.5%) and Inthihas et al. [29] (94.1%) in primary Class II lesions. Higher success rates (100%) were reported by Hendam et al. [59] and Samir et al. [52] in Class V lesions, likely due to differences in clinical assessment criteria (USPHS vs. FDI), cavity location and isolation techniques. FDI criteria are more sensitive, enabling detection of early deterioration in restorations.

Collectively, these findings indicate that while giomer restorations may perform better in certain clinician-assessed esthetic parameters, both materials provide reliable clinical performance and satisfactory patient acceptance over time. This emphasizes that minor statistically significant differences should be interpreted cautiously and within the clinical context, particularly when they do not impact patient perception. Therefore, both restorative materials can be considered viable options in pediatric dentistry, with material selection guided by clinical circumstances and operator preference rather than isolated statistical outcomes.

### Strengths and limitations

This study has several notable strengths. The randomized comparative design allowed for a direct evaluation of two restorative materials (flowable giomer and glass-hybrid-added highly viscous glass ionomer) under the same clinical conditions, thereby reducing variability. Standardized methodology was employed, with all restorations placed according to manufacturers’ instructions by calibrated operator, enhancing internal validity.

The use of contemporary restorative materials (BEAUTIFIL Flow Plus X and EQUIA^®^ Forte HT Fil) ensured that the findings are relevant to current clinical practice. Clinical performance was assessed using validated standardized FDI criteria enabled thorough evaluation of restorations across functional, biological, and esthetic domains. criteria, ensuring objective and reproducible outcome evaluation. The conservative cavity preparation method adhered to contemporary minimally invasive dentistry principles, enhancing the study’s clinical applicability. By focusing on Class II cavities, the research addressed a notable clinical challenge in pediatric dentistry.

However, some limitations must be acknowledged. The sample was drawn from a single clinical setting, which may limit the generalizability of the findings to other populations. Strict inclusion criteria and the exclusion of children requiring advanced behavior management may have led to underrepresentation of more challenging clinical scenarios encountered in everyday pediatric dentistry.

The follow-up period of one year, although sufficient to assess short-term performance, may not capture long-term material durability and failure patterns. Furthermore, the controlled clinical environment in which the treatments were carried out does not fully replicate the variability of real-world practice, where time constraints and varying patient cooperation can influence outcomes.

## Conclusions

This randomized clinical trial provides valuable evidence regarding the comparative clinical performance of BEAUTIFIL Flow Plus X and EQUIA Forte HT Fil in Class II restorations of primary molars over 12 months. Both materials demonstrated acceptable clinical performance, with BEAUTIFIL Flow Plus X showing superior esthetic properties and functional performance while maintaining comparable biological properties. The findings support the use of both materials in pediatric practice, with material selection guided by specific clinical requirements and patient factors.

## Data Availability

The data that support the findings of this study are available on request from the corresponding author. The data is not publicly available due to privacy or ethical restrictions.
